# Effect of Kinesio Taping Combined With Oromotor Stimulation on Feeding Readiness in a Preterm Infant: A Case Report

**DOI:** 10.7759/cureus.111408

**Published:** 2026-06-24

**Authors:** Dhwani D Chanpura, Shrisha Purohit

**Affiliations:** 1 Physiotherapy, College of Physiotherapy, Sumandeep Vidyapeeth Deemed to be University, Vadodara, IND

**Keywords:** feeding difficulties, feeding readiness, kinesio taping, nicu, oral feeding, oromotor stimulation, preterm infant

## Abstract

Feeding difficulties are common in preterm infants due to immature oromotor and neurological systems. Oromotor stimulation (OMS) is widely used, while Kinesio taping (KT) is emerging as a supportive intervention. A preterm male infant born at 35 weeks of gestation presented with poor suck-swallow coordination, reduced tongue mobility, and feeding difficulties in the neonatal intensive care unit (NICU). Baseline assessment included oral intake, weight, and the Early Feeding Skills (EFS) score. A structured seven-day protocol of daily OMS (5 minutes/session) combined with KT applied to the facial and suprahyoid muscles was administered by a trained physiotherapist. Oral intake improved from 432 mL/day to 453 mL/day. Body weight increased from 1.30 kg at baseline to 1.43 kg at the end of the intervention period. The EFS score improved from 57 to 70. No adverse reactions were observed. KT combined with OMS may be a safe and useful intervention for improving feeding readiness in preterm infants.

## Introduction

Feeding difficulties are among the most common developmental challenges encountered in preterm infants and are associated with delayed attainment of oral feeding skills and prolonged hospitalization [1,2]. Successful transition from tube feeding to independent oral feeding requires coordinated maturation of sucking, swallowing, and respiration [1]. However, neurological immaturity, reduced muscle tone, altered sensory processing, and underdeveloped oral structures frequently disrupt this suck-swallow-breathe sequence in preterm neonates [2].

Among the various interventions used to facilitate feeding development, oromotor stimulation (OMS) has gained considerable attention as an evidence-based strategy for improving oral feeding skills in preterm infants. OMS involves structured tactile stimulation of oral structures, including the lips, cheeks, gums, tongue, and jaw, with the aim of enhancing oral sensory awareness, strengthening oral musculature, promoting non-nutritive sucking, and improving coordination of the feeding process. Previous studies have demonstrated that OMS can accelerate the attainment of full oral feeding, improve feeding efficiency, and positively influence feeding readiness among preterm neonates [3,4].

Kinesio taping (KT), originally developed for musculoskeletal rehabilitation, has recently emerged as a potential adjunctive intervention in neonatal practice. Although its exact mechanisms remain incompletely understood, KT is proposed to exert its effects through continuous cutaneous stimulation that enhances proprioceptive input and modulates sensorimotor function. The tactile input generated by the elastic tape may facilitate activation of underlying muscles, improve neuromuscular coordination, and augment sensory feedback. When applied to facial and suprahyoid muscles involved in sucking and swallowing, KT may support the coordinated activity necessary for effective oral feeding [3,5].

Evidence regarding the use of KT in neonatal feeding disorders remains limited but promising. Lin et al. reported improved swallowing function following KT application in a newborn with swallowing difficulties, suggesting that KT may facilitate feeding-related muscle activity. Subsequently, Çelik et al., in a randomized controlled pilot study, demonstrated beneficial effects of KT on swallowing function in newborns experiencing feeding difficulties [4]. More recently, Naderifar et al. described favorable outcomes following the combined use of oral motor interventions and KT in premature infants with feeding problems. Nevertheless, the available literature largely consists of pilot studies and case reports, and robust evidence supporting routine neonatal application of KT is still lacking [6,7].

Sensorimotor interventions have become increasingly integrated into developmental care practices within neonatal intensive care units (NICUs). Approaches aimed at optimizing sensory experiences and promoting neuromotor organization have been associated with improvements in feeding competence and neurobehavioral outcomes in preterm infants. However, despite growing interest in KT as a supportive modality, there remains a paucity of evidence examining its use alongside established interventions such as OMS. In particular, the feasibility, safety, and potential clinical benefits of combining these two approaches to enhance feeding readiness in preterm infants have not been adequately explored.

Therefore, this case report describes the application of combined KT and OMS in a preterm infant with feeding difficulties and examines its potential influence on feeding readiness, oral intake, and short-term clinical outcomes within the NICU setting.

## Case presentation

A late preterm male infant born at 35 weeks of gestation via lower segment cesarean section (LSCS), with a birth weight of 1.3 kg, was admitted to the NICU due to feeding difficulties. The infant had an Apgar score of seven at one minute and nine at five minutes after birth. He was admitted with prematurity, low birth weight, feeding immaturity, and poor oral feeding skills. At the initiation of the intervention, the infant was 12 days old, with a corrected gestational age of 36 weeks and five days, and had a total NICU stay of 19 days. The infant required supplemental oxygen via nasal cannula for the first 48 hours of life, with no subsequent respiratory support. There was no history of respiratory distress syndrome, sepsis, necrotizing enterocolitis, intraventricular hemorrhage, significant neonatal jaundice requiring exchange transfusion, major congenital anomalies, or neurological abnormalities. The infant was receiving routine neonatal supplementation and was not on any medications known to influence feeding performance.

Initial nutritional support was provided through an orogastric tube using expressed breast milk, and oral feeding attempts were gradually introduced according to the infant's tolerance before commencement of the intervention. Clinical assessment revealed weak non-nutritive sucking, reduced jaw excursion, decreased tongue mobility, inconsistent suck-swallow-breathe coordination, occasional breath-holding episodes during feeding attempts, and early fatigue, all of which contributed to poor feeding readiness and impaired oral feeding performance.

A combined intervention protocol consisting of KT and OMS was administered for seven consecutive days by a physiotherapist with over 10 years of experience in neonatal rehabilitation. Prior to the KT application, a 24-hour skin sensitivity test was performed using a small patch of tape applied to the infant's trunk. Considering the fragility of neonatal skin, all tape applications were performed using approximately 15% tension. Kinesio Tex® Gold Light Touch Plus was used throughout the intervention.

**Figure 1:**
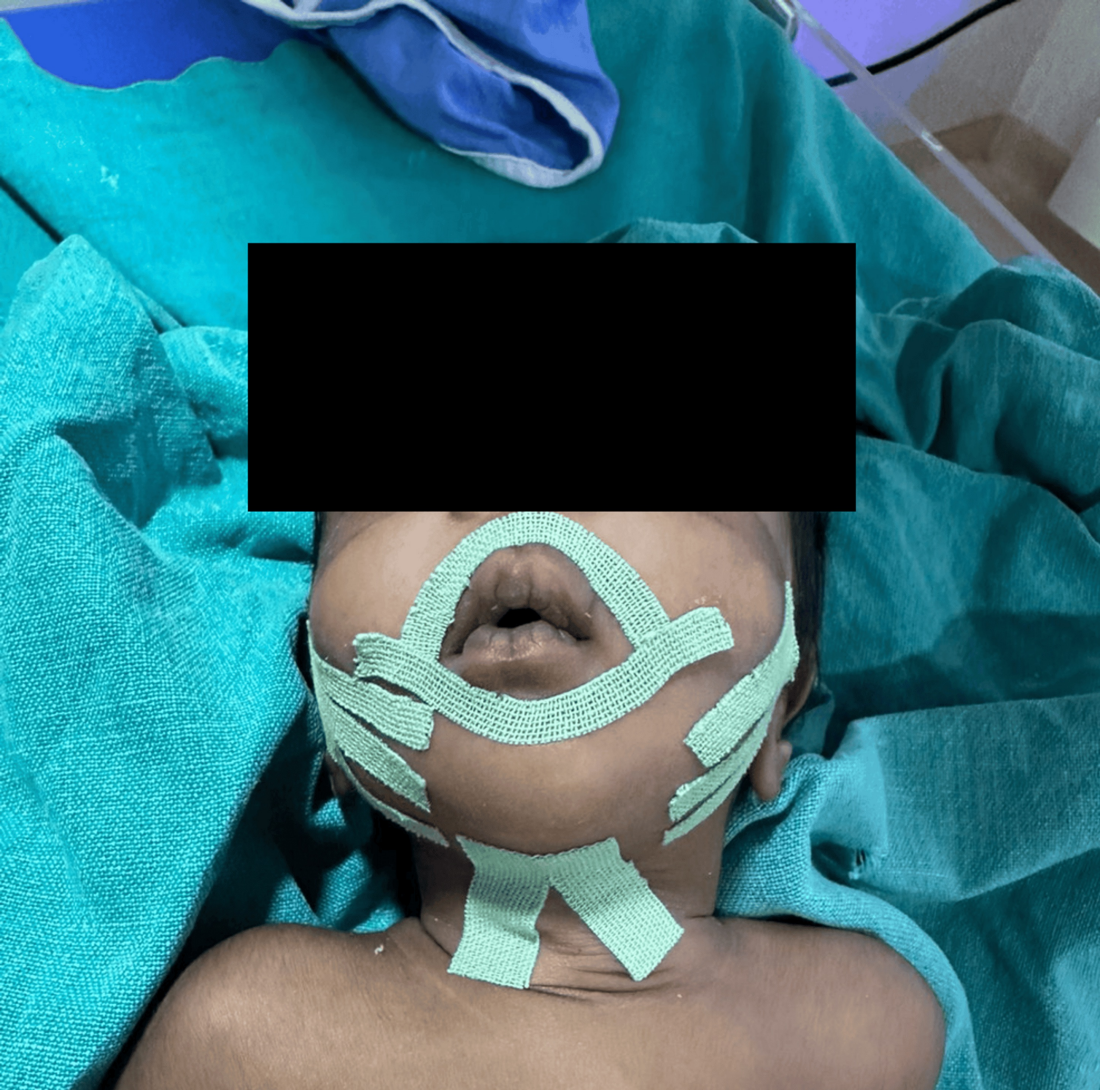
Placement of Kinesio tape

KT was applied to the orbicularis oris, suprahyoid, and masseter muscles to facilitate lip closure, swallowing, and jaw movement, respectively. Two I-strips were applied over the upper and lower orbicularis oris muscles, one Y-strip was used over the mylohyoid and sternohyoid regions to support hyoid elevation during swallowing, and a three-tailed strip was applied over the masseter muscle to improve jaw stability (Figure 1). The tape remained in place for 48 hours and was reapplied using the same technique, resulting in a total of four applications during the seven-day intervention period.

Immediately following the KT application, OMS was administered once daily for five minutes, five to 15 minutes before feeding, following a structured protocol adapted from Fucile et al. Gentle tactile stimulation was sequentially applied to the cheeks, lips, gums, tongue, and jaw to enhance oral sensory awareness, improve non-nutritive sucking, facilitate suck-swallow-breathe coordination, and promote feeding readiness.

Outcome measures

Feeding performance was assessed using the Early Feeding Skills (EFS) Assessment Tool, with higher scores indicating better feeding competence. Outcome measures included EFS scores, total daily enteral feeding intake, and body weight, assessed at baseline and after completion of the seven-day intervention period. The reported feeding intake values represented the infant's total daily enteral intake rather than oral intake alone.

## Discussion

This case describes the feasibility and tolerability of combining KT with OMS in a preterm infant with feeding difficulties, with improvements observed in feeding readiness, feeding performance, oral intake, and weight gain during the intervention period. KT may support oral feeding by providing cutaneous sensory input and enhancing neuromuscular activation and coordination of the structures involved in sucking and swallowing. The observed improvements are consistent with previous reports describing positive effects of sensorimotor interventions on feeding outcomes in preterm infants [5-7].

The combined intervention was safe, feasible, and well-tolerated in the NICU setting, with no adverse skin reactions, cardiorespiratory instability, or other intervention-related adverse events observed throughout the treatment period. However, as KT and OMS were administered concurrently, the individual contribution of each intervention could not be determined. Furthermore, the observed improvements may also reflect normal neurological maturation, advancement of nutritional support, and routine NICU care. Therefore, causality cannot be inferred from this single-case design.

Additional controlled studies with larger sample sizes are needed to establish the effectiveness of KT as an adjunct to conventional feeding interventions and to further explore the potential role of combined sensorimotor approaches in supporting feeding outcomes in preterm infants.

## Conclusions

This case demonstrates that the combined use of KT and OMS was feasible, well-tolerated, and safely implemented in a preterm infant with feeding difficulties in the NICU setting. Improvements in feeding readiness, feeding performance, and weight gain were observed during the intervention period. However, the independent effects of KT cannot be determined, and the contribution of normal maturation, nutritional progression, and concurrent NICU care cannot be excluded. Therefore, efficacy cannot be inferred from this single case report, and further controlled studies are required to evaluate the potential role of this combined intervention in supporting feeding outcomes in preterm infants.
